# Mechanism to control the cell lysis and the cell survival strategy in stationary phase under heat stress

**DOI:** 10.1186/s40064-015-1415-7

**Published:** 2015-10-13

**Authors:** Rashed Noor

**Affiliations:** Department of Microbiology, Stamford University Bangladesh, 51 Siddeswari Road, Dhaka, 1217 Bangladesh

**Keywords:** *Escherichia coli*, Heat stress, σ^H^, σ^E^, Programmed cell death (PCD), Heat shock proteins (HSPs)

## Abstract

An array of stress signals triggering the bacterial cellular stress response is well known in *Escherichia coli* and other bacteria. Heat stress is usually sensed through the misfolded outer membrane porin (OMP) precursors in the periplasm, resulting in the activation of σ^E^ (encoded by *rpoE*), which binds to RNA polymerase to start the transcription of genes required for responding against the heat stress signal. At the elevated temperatures, σ^E^ also serves as the transcription factor for σ^H^ (the main heat shock sigma factor, encoded by *rpoH*), which is involved in the expression of several genes whose products deal with the cytoplasmic unfolded proteins. Besides, oxidative stress in form of the reactive oxygen species (ROS) that accumulate due to heat stress, has been found to give rise to viable but non-culturable (VBNC) cells at the early stationary phase, which is in turn lysed by the σ^E^-dependent process. Such lysis of the defective cells may generate nutrients for the remaining population to survive with the capacity of formation of colony forming units (CFUs). σ^H^ is also known to regulate the transcription of the major heat shock proteins (HSPs) required for heat shock response (HSR) resulting in cellular survival. Present review concentrated on the cellular survival against heat stress employing the harmonized impact of σ^E^ and σ^H^ regulons and the HSPs as well as their inter connectivity towards the maintenance of cellular survival.

## Background

An assortment of physicochemical stress stimuli triggering the cellular defense related stress responsive mechanisms have been identified so far in bacteria, largely in *Escherichia coli*, and to certain extent in other microorganisms (Franchini et al. 2015; Munna et al. 2015; Nur et al. 2014; Nagamitsu et al. 2013; Murata et al. 2012; Valdez-Cruz et al. 2011; Rudolph et al. 2010; Caspeta et al. 2009; Noor et al. 2009a, b; Kim et al. 2007; Guisbert et al. 2007; Raivio and Silhavy 2000; Nitta et al. 2000; Hengge-Aronis 2000). The principal stress signals include nutrient exhaustion, elevated temperature, alteration in pH and the redox state, variations in salt concentrations, increased amount of internal reactive oxygen species (ROS), external oxidants like hydrogen peroxide (H_2_O_2_), other toxic chemicals, etc. (Munna et al. 2015; Nur et al. 2014). Such stress signals in *E*. *coli* are usually sensed by the increase in the outer membrane porin (OMP) precursors in the periplasm, which are further transduced into the cytoplasm resulting in the activation of the genes necessary for the cellular homeostatic recovery (Shenhar et al. 2009; Hayden and Ades 2008; Kim et al. 2007).

In bacteria, many stress responses are generated by the alternative sigma factors that can rapidly reprogram the necessary gene expression against various stress signals by recruiting RNA polymerase to specific subsets of stress responsive promoters in the cell (Campagne et al. 2015; Paget 2015; Murata et al. 2012; Kim et al. 2007; Gruber and Gross 2003). So far seven sigma factors have been identified that differently recognize about 2000 promoters on the *E*. *coli* genome to express around 4300 genes (Jin et al. 2013; Ishihama 1999). These factors include σ^D^ (σ^70^, the “housekeeping” sigma factor, encoded by *rpoD*), σN (σ^54^, the nitrogen-limitation sigma factor, encoded by *rpoN*), σ^S^ (σ^38^, the stationary phase sigma factor, encoded by *rpoS*), σ^H^ (σ^32^, the heat shock sigma factor, encoded by *rpoH*), σ^F^ (σ^28^, the flagellar sigma factor, encoded by *rpoF*), σ^E^ (σ^24^, the extracytoplasmic sigma factor, encoded by *rpoE*), and σ^FecI^ (σ^19^, the ferric citrate sigma factor, encoded by *fecI*). Interestingly, an extensive functional overlap has been noticed between the σ factors: the majority of σ^H^ promoter targets overlap with those of σ^D^, and σ^E^ regulated promoters also overlaps extensively with those for σ^D^ (Wade et al. 2006). In *E. coli* the stress caused by elevated temperatures has long been known to be regulated by two alternative sigma factors, σ^H^ (encoded by *rpoH*) and σ^E^, governing the transcription of two respective heat-shock regulons to cope with protein misfolding in the cytoplasm and the extra-cytoplasm (periplasm and outer membrane), respectively (Dartigalongue and Raina 1998; Raina et al. 1995).

Indeed, a number of reports very clearly unravelled the cellular defence strategies triggered by heat stress. Present review simply compiled the information regarding the heat shock response in *E. coli* and attempted to decipher the cell survival strategies employing the sigma factors and the chaperon proteins at high temperature. The interesting part of the present review would be the aspects of correlation of the ROS concentrations with the formation of viable nut nonculturable (VBNC) cells triggering the induction of their lysis together with a possible cellular survival output.

## Cell survival employing heat-shock proteins (HSPs) in concert with sigma factors

The transcription of the *rpoH* gene for σ^H^ is induced at elevated temperature via the action of σ^E^ (Erickson and Gross 1989). σ^E^ is in part regulated by a cognate small RNA (as discussed later), and σ^H^ synthesis is regulated by structural change of its own mRNA molecules serving as a cellular thermometer and its activity modulated by phosphorylation (Klein et al. 2003). In *E*. *coli*, around 400 genes have been reported to be up-regulated by the transient heat shock by up-shifting the incubation temperature from 37 to 43 °C (Gunasekera et al. 2008). Indeed, to deal with high temperature stress in *E. coli*, GroEL and DnaK protein amounts are largely elevated (Morimoto 2012; Morimoto et al. 2011; Guisbert et al. 2008; Kedzierska 2005). σ^H^ is principally known to regulate the transcription of the major heat shock proteins (HSPs) and molecular chaperons required for heat shock response (HSR) resulting in cellular survival. Transcription of the *rpoH* gene for σ^H^ is induced at elevated temperature via the action of σ^E^ (Lim et al. 2013; Murata et al. 2011). The control of the expression of HSPs has been found to be highly variable among different bacteria (Gonzalez et al. 2013; Urban-Chmiel et al. 2013; Stephanou and Latchman 2011; Raina et al. 1995). Most of the HSPs including GroEL and DnaK, ATP-dependent proteases of Lon, HslUV, Clp and FtsH (HflB), periplasmic protease DegP, etc. are already known to be involved in protein folding, refolding, quality control and degradation, removal of damaged proteins, and are induced in response to stress (Ryabova et al. 2013; Murata et al. 2011; Gunasekera et al. 2008; Mcbroom and Kuehn 2007; Kabir et al. 2005; Ades 2004; Arsene et al. 2000; Missiakas et al. 1997).

Protein folding is known to be mediated principally by the ribosome-associated trigger factor (TF), the Hsp70 system DnaK/DnaJ/GrpE and the chaperonin system GroES/GroEL (Kumar and Sourjik 2012). While DnaK/DnaJ/GrpE is the most adaptable chaperone system in *E. coli*, and GroEL and its cofactor GroES are known to be essential for cell viability (Hartl et al. 2011), another highly conserved chaperone system Hsp90 (HtpG in *E. coli*) is still known to be not that functional (Kumar and Sourjik 2012). Hsp100 (ClpB in *E. coli*) and some other small HSPs are primarily involved in refolding of the unfolded or aggregated proteins (Goeser et al. 2015; Kumar and Sourjik 2012). Interestingly, most chaperone systems are co-localized to the heat-induced protein aggregates in *E. coli* (Winkler et al. 2010). Besides, periplasmic protease DegP, the CpxAR (Cpx) mechanism, involvement of BaeRS (Bae), and the events of Rcs phosphorelays, the phage shock (PSP) response, and the responses generated by ppGpp in response to heat shock become functional (Alexopoulos et al. 2013; Barchinger and Ades 2013; Kumar and Sourjik 2012; Morimoto 2012; Suzuki et al. 2012; Majdalani and Gottesman 2005; Raivio 2005; Rowley et al. 2006; Ruiz and Silhavy 2005; Artsimovitch et al. 2004; Porankiewicz et al. 1999).

The controlling modulators of σ^H^ are the DnaK chaperone system together with the metallo-protease FtsH (HflB) (Arsene et al. 2000; Straus et al. 1987; Bukau 1993). Afterward the up-regulation of genes encoding the HSPs during the increase of temperature has been intensely investigated (Morimoto 2012; Valdez-Cruz et al. 2011; Akerfelt et al. 2010; Klein et al. 2003; Guisbert et al. 2004, 2008; Yura et al. 2007; Genevaux et al. 2007; Georgopoulos 2006; Wade et al. 2006; Weber et al. 2005; Gruber and Gross 2003). In order to combat against the permanently changing environmental conditions including heat shock, *E. coli* has been recently reported to employ ClpXP, ClpAP, HslUV, Lon and FtsH, for the regulated proteolysis which is required to adjust the cellular protein pool (Bittner et al. 2015). Moreover, in response to heat shock, ATP-driven proteolysis by the Clp protease (Clp/Hsp100 chaperone family) has been reported to play a vital role in the removal of non-functional damaged proteins which is indeed in favor of cell homeostasis (Alexopoulos et al. 2013; Porankiewicz et al. 1999; Gottesman 1996).

σ^S^, which is encoded by the *rpoS* gene, functions as the master regulator of the general stress response, and can be activated within the range of temperature <37–41 °C (King et al. 2014). σ^D^ is usually activated during the maximal cellular growth especially at 37–41 °C. During the log phase, cells remain viable with the potential of colony formation. When the reactive oxygen species (ROS) accumulates and transforms the culturable cells into viable but nonculturable (VBNC) form, cells undergo stasis of which a major fraction undergoes σ^E^-dependent lysis (Noor et al. 2009b). Upon heat shock (>42 °C), most cells become non-culturable, and the *rpoE*-encoded alternative sigma factor σ^E^ and the *rpoH*-encoded classical heat‐shock sigma factor σ^H^ act as the regulator of the extra-cytoplasmic HSPs (Chakraborty et al. 2014; Morimoto 2012; Murata et al. 2011).

**Fig. 1:**
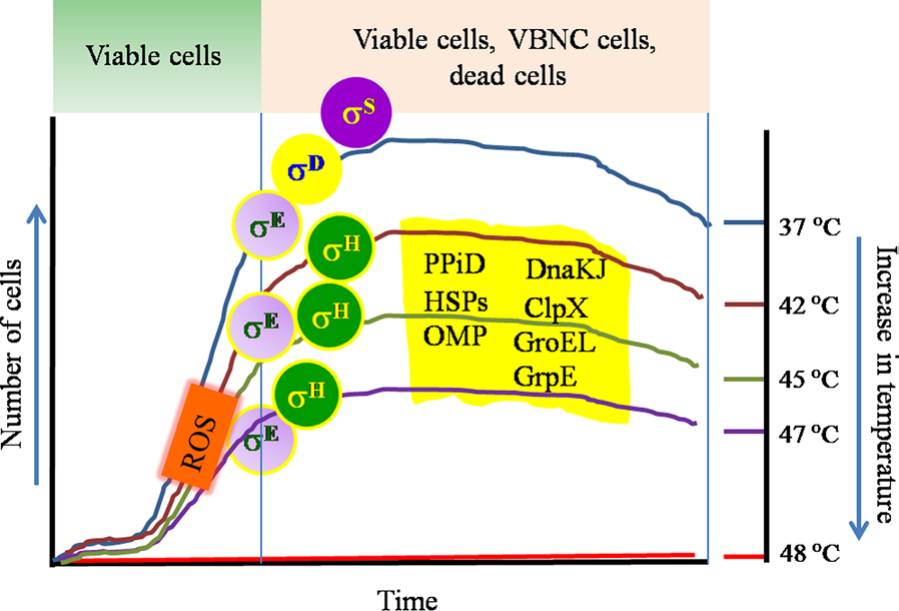
Activation of chaperon system and their regulating sigma factors in *Escherichia coli* cells due to heat shock. *rpoS* encoded alternative sigma factor σ^S^, which acts as the master regulator of the general stress response is usually activated at the early stationary phase at any temperature (even below 37 °C). Up to the early stationary phase, usually cells are observed as viable and culturable (as shown in *green box*); however, during the entry into the stationary phase, a significant fraction of the total cell population become viable but nonculturable (VBNC) due to the accumulation of the reactive oxygen species (ROS). The figure shows the possible recruitment of the heat shock proteins (HSPs) in association of different sigma factors under heat stress. The *rpoD* encoded housekeeping sigma factor or the primary sigma factor σ^D^ together with σ^S^ transcribing most of the genes in growing cells are activated at 37–41 °C, a temperature range where cells are presumably found at their maximal growing state. Transcription of the *rpoH* gene for σ^H^ is induced at elevated temperature (generally at 42 °C or more) via the action of σ^E^. Both σ^E^ and σ^H^ act as the regulator of the extra-cytoplasmic/extreme HSPs (DnaKJ, OMP, PPiD, GroEL, ClpX, GrpE). The HSPs are expected to be activated at 42–48 °C, where cells are usually completely non-culturable. Correlated reference of the narrated temperatures can be found in the text

Besides, PpiD, the outer membrane porin, (OMP) biogenesis factor with a robust peptidyl-prolyl isomerase activity, is encoded by *ppiD* gene which belongs to two stress regulons: the CpxR–CpxA regulon and the σ^H^ regulon (Noor et al. 2009a; Dartigalongue and Raina 1998). This is also to be mentioned that the Cpx system in cohort with σ^E^ monitor the stress signals in the cell envelope in *E. coli* (Matern et al. 2010; Ruiz and Silhavy 2005). Thus the possible recruitment of the HSPs by the different sigma factors, involvement of ROS generated by heat stress, and the subsequent defect in the OMP biogenesis in relation to the PpiD activity within a range of temperatures would be interesting to decipher the heat shock events in *E. coli* cells (Fig. 1).

## σ^E^-dependent programmed cell death (PCD) as the cell survival strategy

The physiological changes in cellular behaviour and the association of sigma factors in the stress responsive events have been elaborately noticed earlier (Murata et al. 2012; Noor et al. 2009a; Erickson and Gross 1989; Raina et al. 1995). Indeed at the early stationary phase, *E*. *coli* cells have been noticed to undergo a decrease in the number of viable cells, and as the stationary phase progresses, interestingly cells keep sustaining their colony-forming abilities (Zambrano et al. 1993). However, at the entry of the stationary phase, in parallel with the decline in colony forming units (CFUs), cells that are viable but defective in the formation of CFUs; i.e., viable but nonculturable (VBNC) cells (Cuny et al. 2005; Desnues et al. 2003; Nystrom 2003; Nitta et al. 2000), tend to accumulate at the early stationary phase, and undergo lysis with a concomitant increase in the amounts of σ^E^ (Nitta et al. 2000; Kabir and Yamada 2005). Interestingly, such lysis has been noticed to remove the damaged cells (supported by the reduction in cell turbidity at an optical density of 600 nm or at OD_600_) but have no or minor impact on the cellular potential of formation of colonies (Noor et al. 2009a, b; Kabir et al. 2004, 2005; Nitta et al. 2000). Thus, in consistent to the earlier hypothesis (i.e., VBNC cells typically demonstrate decreased metabolic activity while on resuscitation they become culturable), the VBNC cells may be considered to define a specific program of differentiation into a long-term survival state (Oliver 2005; Bogosian and Bourneuf 2001; Villarino et al. 2000). In this context, the specific lysis of VBNC cells may be considered as the programmed cell death (PCD), with the elucidation of the mechanism of σ^E^-directed PCD (Noor et al. 2009a; Nitta et al. 2000). This mechanism might be physiologically important for *E*. *coli* because it can eliminate specifically the VBNC cell population.

**Fig. 2:**
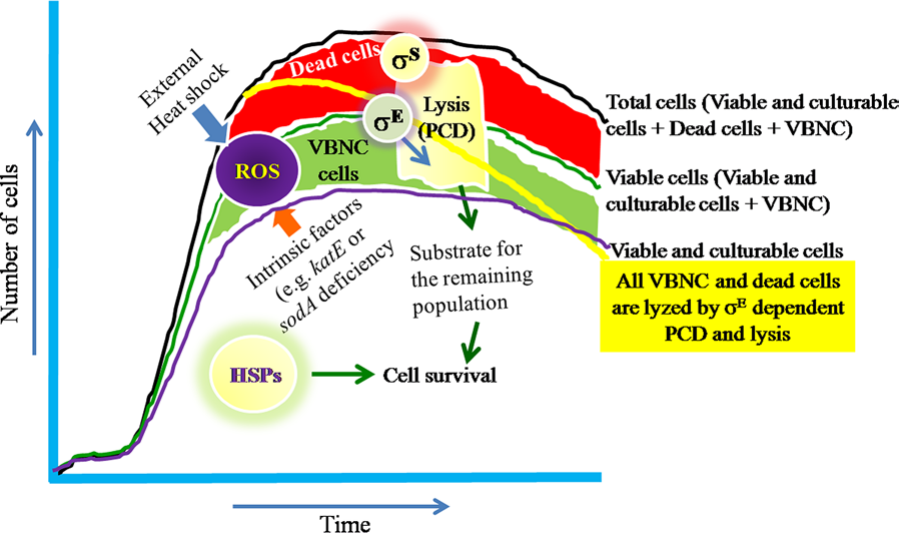
Survival strategy of stressed *Escherichia coli* cells employing σ^E^-directed cell lysis. The figure denoted the dead cells as the *red fraction* and the viable but non-culturable (VBNC) cells as *green.* Upon entry into the stationary phase, the stressed cells of *E. coli* are transformed into VBNC state and undergo lysis with a concomitant increase in the amounts of alternative sigma factor σ^E^ (i.e., the σ^E^-directed programmed cell death, PCD), as shown by the *yellow line*. Optimistically, this mechanism might be important for stressed cells of *E*. *coli* because the extracellular fractions of lysed cells might act as nutritional supplement for the survival of the remaining population

Moreover, during survival especially under nutrient starvation, organic substances (proteins, dissolved free amino acids, and dissolved monomeric carbohydrates) are released into the surrounding medium of *E. coil* with a concomitant transition from the culturable state to the VBNC state (Arana et al. 2004). Thus, it was suggestive that the organic molecules released into the medium play a role in the transition from culturable to VBNC state. The mutation of the *rpoS* gene encoding σ^S^ is also known to cause an increase in VBNC cells (Kabir et al. 2004). Afterward a novel point was suggested that the cellular excretion materials could be used as nutrients for the remaining population as both the culturable cells and VBNC cells were noticed in the stationary phase by Noor et al. 2009a (Fig. 2). Interestingly a culturable population was observed during stasis even upon prolonged incubation revealing the cell survival trait which may support such cell survival strategy (Noor et al. 2009a). Consistently, as stated by Murata et al. 2012, since most of cells (~60 %) become VBNC in the early stationary phase, the σ^E^-directed cell lysis would eventually contribute to the removal of these damaged cells and the resultant aggregates may serve as nutrients to remaining living cells.

## Functional analysis of *rseA*, *rseB*, *rseC* genes in cell survival

**Fig. 3:**
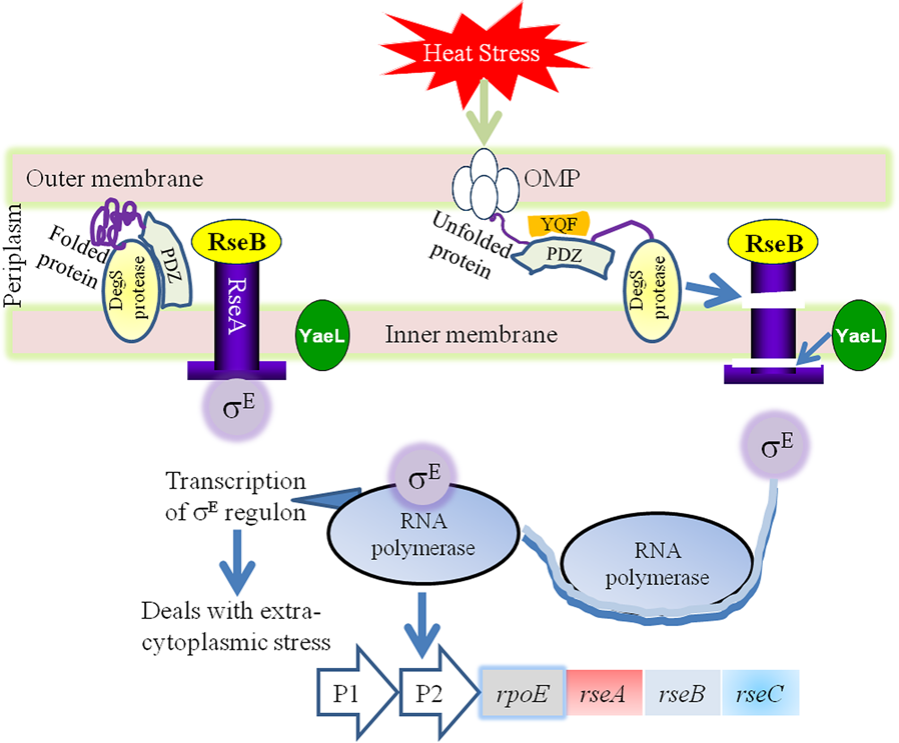
Functional model of *rpoE*-*rseABC* operon and role of σ^E^ in combating heat stress. The *rseA* gene product, RseA functions as an anti-sigma factor by binding to σ^E^ under normal condition. RseB also acts as a negative modulator and keep attached to RseA. Thus, the association of σ^E^ with RNA polymerase is hindered by formation of a tight complex between σ^E^ and RseA. Under stress condition, the YQF motif of the PDZ domain of the DegS protease senses the stress signal (usually through protein misfolding; i.e., the misfolded OMP precursor), RseB gets detached from RseA followed by the cleavage of C-terminus domain of RseA by DegS, and the further proteolytic cleavage of RseA by the metalloprotease YaeL results in the liberation of σ^E^ from RseA. The free σ^E^ binds to RNA polymerase which starts the transcription of the *rpoE*-*rseABC* operon genes required for response against stress signal

**Fig. 4:**
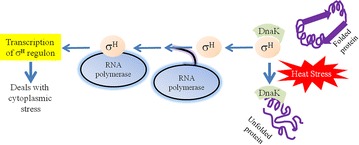
Function of DnaK in association with σ^H^ in response to heat stress. Unfolded proteins caused by heat stress are sensed by the heat shock chaperon DnaK which keeps attached to σ^H^. Upon protein unfolding, DnaK releases σ^H^ which in turn binds to RNA polymerase. The σ^H^—RNA polymerase complex transcribes genes whose products are essential to respond against the cytoplasmic stress. This is to be mentioned that the transcription of the σ^H^ (encoded by the *rpoH* gene) is induced at elevated temperature via the action of σ^E^ (encoded by the *rpoE* gene)

One of envelope-stress responses in *E*. *coli* is performed by a regulated intra-membrane proteolysis system (Barchinger and Ades 2013; Cezairliyan and Sauer 2006) that includes the σ^E^ transcription factor, the RseA and RseB regulators, and the DegS and RseP (YaeL) proteases (Noor et al. 2009a; Cezairliyan and Sauer 2006; Kabir and Yamada 2005; Ruiz and Silhavy 2005; Ades 2004; Alba and Gross 2004; Ehrmann and Clausen 2004; Duguay and Silhavy 2004; Campbell et al. 2003). The association of σ^E^ with RNA polymerase is normally inhibited by formation of a tight complex between σ^E^ and RseA (Fig. 3). At high temperature or under other conditions that result in protein misfolding, a series of proteolytic cleavages destroy RseA and liberate σ^E^ in free form (Alba et al. 2002). DegS, which is anchored into the inner membrane by an N-terminal transmembrane segment, is activated when the PDZ domain of DegS binds to a misfolded OMP precursor (Kim et al. 2007; Ades et al. 1999). The periplasmic C-terminus of RseA is then cleaved by the activated DegS (Wilken et al. 2004), followed by the sequential digestion of the N terminus of RseA by RseP (YaeL), a membrane metalloprotease, to release the N terminus of RseA/σ^E^ complex into the cytosol (Kanehara et al. 2002). The σ^E^ can be released completely from RseA, and its activity as a sigma factor is restored when the RseA fragment is removed by ClpXP protease (Kim et al. 2007; Flynn et al. 2004). The models shown in Figs. 3, 4 specifically demonstrated that the σ^E^ regulon genes deal with extracytoplasmic stress, whereas the σ^H^ regulon genes deal with the cytoplasmic stress (Kabir and Yamada 2005).

## Role of *sodA* and *katE* genes and the trigger of σ^E^-dependent cell lysis

**Fig. 5:**
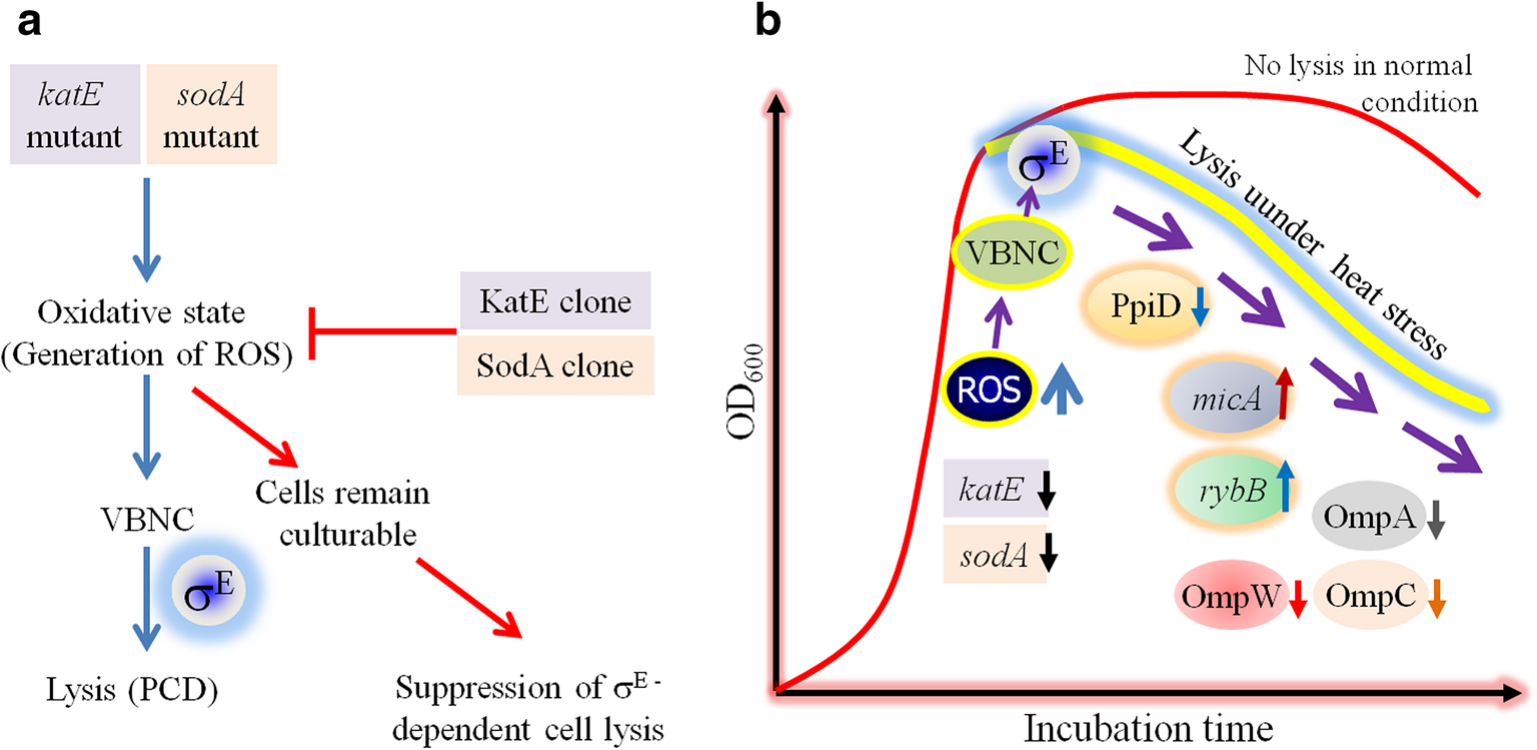
Generation of heat shock and the consequent mechanism of σ^E^-dependent cell lysis. **a** Functional analysis of *sodA* and *katE* genes, involved in the evolution of reactive oxygen species (ROS). Mutation in *sodA* (encoding superoxide dismutase) and *katE* (encoding catalase) induces the generation of ROS which may render the viable and culturable cells to be damaged or into the VBNC state, which in turn undergoes the σ^E^-directed lysis. Thus the defective cells are removed and only the culturable cells sustain thereby suggesting the phenomenon of programmed cell death (PCD). The idea is supported by overexpressing the *sodA* (*SodA clone*) and *katE* genes (*KatE clone*) whereby the ROS accumulation is considerably suppressed with a concomitant downregulation of the *rpoE* gene (encoding σ^E^). **b** Possible mechanism of σ^E^-dependent cell lysis whereby the role of ROS has been shown in the formation of the VBNC cells which have further undergone the lysis process with a concominant increased downregulation of peptidyl prolyl isomerise (PpiD) and the simultaneous upregulation of the *micA* and *rybB* genes encoding small RNAs. The repression of the expression of the outer membrane porins (OMPs) have also been detected which might be responsible for the impairment of the cell membrane integrity thereby rendering the cells to be lyzed

Afterward, on the basis of the suggestive data of accumulation of oxidative stresses (in the form of reactive oxygen species; i.e., ROS) at the early stationary phase (Desnues et al. 2003; Dukan and Nystrom 1999) to cause formation of VBNC cells (Cuny et al. 2005; Nystrom 2005a, b), the trigger of the lysis process was analyzed using the deletion mutant of *katE* (encoding catalase) and by over-expressing the *sodA* (encoding superoxide dismutase) and *katE* genes (Noor et al. 2009b). The deletion mutant of *katE* was noticed to result in the elevation of the amounts of intracellular ROS with a concomitant increase in VBNC cells which were further subjected to typical σ^E^-dependent cell lysis (Noor et al. 2009b). Both the amount of ROS and cell lysis level were found to be significantly reduced by the overexpression of *sodA* and *katE* genes (Fig. 5a). Thus, together with the knowledge of roles of HSPs under the control of σ^E^ and σ^H^ regulons (as stated earlier), the discovery of the trigger of the lysis of both VBNC and the damaged cells of *E. coli* led to model the total survival mechanism under stresses condition (Figs. 2, 4 and 5a).

## Heat shock and evolution of oxidative stress (through ROS generation)

The influence of the temperature up-shift on the generation of intracellular oxidative stress has been detected earlier (Noor et al. 2013; Yamada et al. 2009), and the impulsive accretion of the ROS at the early stationary phase of bacterial growth has been monitored (Yamada et al. 2009). As stated above, our earlier study (Noor et al. 2009b) has shown that mutation in *sodA* and *katE* genes induced the elevated accumulation ROS, resulting in the formation of VBNC cells state, which ultimately underwent the σ^E^-directed cell lysis (Fig. 5a). Consistently when these genes were overexpressed, the ROS accumulation was noticed to be suppressed in association to the repression of the σ^E^ level (Noor et al. 2009b). The role of ROS as the trigger of such lysis involving the σ^E^ regulon small RNAs and the subsequent defect in OMP biogenesis proteins will be discussed later (Fig. 5b).

## Role of PPiD, small RNAs and OMP in σ^E^-dependent cell lysis

While a number of ATP-dependent chaperones exist in the cytoplasm, the periplasm harbors two defined types of folding catalysts: protein disulfide isomerase (PDI) and peptidyl–prolyl *cis*–*trans* isomerase (PPIase) (Dartigalongue and Raina 1998). PPIases (PpiA, PPiD and FkpA and SurA of the parvulin family in the periplasm, and PPiC of the parvulin family in the cytoplasm) are known to catalyse the rapid interconversion between the *cis* and *trans* forms of the peptide bond Xaa–Pro (Dartigalongue and Raina 1998). PPiD is known to be the first member of a periplasmic folding catalyst which is regulated by the classical heat-shock sigma factor σ^H^ whereas SurA has a general chaperone-like function involved in correcting the misfolded OMP monomers whose accumulation induce the σ^E^-dependent response in the extra-cytoplasm (Lazar and Kolter 1996; Rouviere and Gross 1996).

As stated earlier, a reduction in the amounts of PpiD in the strain possessing the σ^E^-depenent cell lysis was observed (Noor et al. 2009a). Consistently, suppression of such lysis was observed upon increased expression of the *ppiD* gene (encoding PpiD), with the OMP folding function. Moreover, the increased expression of the *rpoE* gene (encoding σ^E^) has been shown to reduce the reduction in the levels of OMPs (Murata et al. 2012). Indeed, PpiD is known to recognize the early OMP folding intermediates, and hence its over-expression suppresses OMP biogenesis defects. As stated above, the levels of PpiD were found to be severely reduced in the *rseA* mutants (with an increased frequency of σ^E^-depenent cell lysis) (Noor et al. 2009a). Such a reduction in the levels of PpiD could partly account for OMP reduction and hence cell lysis in Δ*rseA* mutants. Therefore, the cell lysis phenotype was evidently deciphered due to reduction in the amounts of major OMPs, and conversely, the PpiD over-expression could be the cause of suppression of this lysis phenotype due to acceleration of OMP folding (Fig. 5b). The findings thus revealed an innate mechanism of cell lysis associated with the integrity of the outer membrane (OM), which is apparently impaired in the *rseA* mutants (Murata et al. 2012).

A second aspect of the σ^E^ response may involve the small Hfq-binding RNAs (two σ^E^-dependent small noncoding RNAs (sRNAs), MicA and RybB or Hfq chaperon) which play a major role in maintaining envelope homeostasis, rapid removal of multiple *omp* transcripts in response to elevated activity of the alternative sigma factor (Peng et al. 2014; Johansen et al. 2008; Vogel and Papenfort 2006). Indeed an ingenious investigation conducted by our group (Murata et al. 2012) on the consequence of sRNA (MicA and RybB, as the respective genes were under the control of σ^E^ regulon as well as the small RNAs being regulators of outer membrane protein, *omp* genes) on the σ^E^-dependent lysis process. Among the *omp* gene products, OmpA is known to be involved in the protection of cell shape and nutrient passage through the outer membrane, OmpC serves as the principal cation-selective porin, while OmpW is assumptive of conferring the cellular integrity (Murata et al. 2012). The *micA*- and *rybB*-disrupted mutations were found to completely repress the cell lysis. Interestingly the increased expression of *micA* and *rybB* genes or the disrupted mutants of *ompA*, *ompC* and *ompW* was noticed to enhance the cell lysis. Experimental demonstration by other studies also showed that the transient induction of RybB resulted in the reduced amounts of mRNA transcripts encoding OmpC and OmpW (Johansen et al. 2006). Mutation in the *rybB* gene resulted in the abolition of the σ^E^-mediated regulation of *ompC* and *ompW* (Johansen et al. 2006). Thus the sRNA-OMP network is of physiologically importance whereby some sRNAs act specifically on a single *omp* mRNA, whereas others control the multiple *omp* mRNA targets (Vogel and Papenfort 2006).

The possible mechanism of σ^E^-dependent lysis with the involvement of sRNA with subsequent on Omp proteins has been modelled in Fig. 5b. The model shows that the activation of σ^E^ stimulates the expression of *micA* and *rybB* genes (encoding the respective small RNAs) with a concomitant reduction in Omp proteins, which in turn causes the disintegration of the outer membrane finally resulting in cell lysis. This is to be mentioned that the expressional control of *omp* genes (encoding OmpC, OmpA and OmpW of outer membrane proteins) is achieved by small RNAs of *micA* and *rybB*, of which genes are strictly under the control of σ^E^ (Valentin-Hansen et al. 2007). Interestingly MicA and RybB were found to be directly involved in cell lysis under ordinary growth conditions in the wild type strains of *E. coli*; i.e., when σ^E^ is not expressed (Murata et al. 2012). One of the possible signals for conversion of colony-formable cells to VBNC cells was identified as the accumulation of intracellular reactive oxygen species (ROS) around the beginning of stationary phase generating oxidative stress (Noor et al. 2009b). Mutations in both *rpoS* and *katE* was found to induce σ^E^-dependent cell lysis (Noor et al. 2009b; Kabir et al. 2004). Moreover, the mutation of *sodA* (encoding superoxide dismutase) was found to significantly increase the expression of the *rpoE* gene encoding σ^E^ (Noor et al. 2009b). Thus the involvement of ROS (either normally or due to the mutation of *katE* and *sodA* genes) has been shown in this model. Noor et al. 2009a showed that the PpiD (the OMP biogenesis factor) amounts were uniquely reduced when σ^E^ levels were elevated. Such reduction in the levels of PpiD thus seems to reflect the cell lysis phenotype. Hence the PpiD has been included in Fig. 5b.

## Conclusion

The present review comprehended the role of sigma factors, the heat shock proteins (HSPs), and the possible interaction between them in combating the cellular stress in *E. coli* evoked by the heat shock. The cellular events in the early stationary phase have been clearly discussed during the heat stress in terms of the elevation impact of the reactive oxygen species (ROS). Accumulation of lysis protein in course of cell incubation has been correlated with the stress resistance mechanisms. Roles of outer membrane porin (OMP), small RNAs, PPiD have clearly been demonstrated to disrupt the cell membrane integrity upon the σ^E^-directed lysis of the viable but non-culturable (VBNC) cells accumulated due to the increased ROS level due to heat stress.
